# Hospital Note

**Published:** 1906-04

**Authors:** 


### HOSPITAL NOTE.

DR. LILLIAN CRAIG RANDALL has enlarged and improved the operating room at her Riverside Hospital. Recent changes in the administrative force of the hospital are: Dr. H. C. Moore of Toronto is now house surgeon, and Miss Anna Conlon is superintendent of nurses.

